# Akinetic swept-source optical coherence tomography based on a pulse-modulated active mode locking fiber laser for human retinal imaging

**DOI:** 10.1038/s41598-018-36252-z

**Published:** 2018-12-05

**Authors:** Hwi Don Lee, Gyeong Hun Kim, Jun Geun Shin, Boram Lee, Chang-Seok Kim, Tae Joong Eom

**Affiliations:** 1 Advanced Photonics Research Institute, Gwangju Institute of Science and Technology, Gwangju, 61005 South Korea; 2 Department of Cogno-Mechatronics Engineering, Pusan National University, Busan, 46241 South Korea; 3 Department of ophthalmology, Korea University college of medicine, Seoul, 02841 South Korea

## Abstract

Optical coherence tomography (OCT) is a noninvasive imaging modality that can provide high-resolution, cross-sectional images of tissues. Especially in retinal imaging, OCT has become one of the most valuable imaging tools for diagnosing eye diseases. Considering the scattering and absorption properties of the eye, the 1000-nm OCT system is preferred for retinal imaging. In this study, we describe the use of an akinetic swept-source OCT system based on a pulse-modulated active mode locking (AML) fiber laser at a 1080-nm wavelength for *in-vivo* human retinal imaging. The akinetic AML wavelength-swept fiber laser was constructed with polarization-maintaining fiber that has an average linewidth of 0.625 nm, a spectral bandwidth of 81.15 nm, and duty ratio of 90% without the buffering method. We successfully obtained *in-vivo* human retinal images using the proposed OCT system without the additional k-clock and the frequency shifter that provides a wide field of view of 43.1°. The main retina layers, such as the retinal pigment epithelium, can be distinguished from the OCT image with an axial resolution of 6.3 μm with this OCT system.

## Introduction

Optical coherence tomography (OCT) is a noninvasive imaging modality that can provide high-resolution, cross-sectional images of tissues^1–3^. OCT has evolved over the past decades as one of the most important clinically applicable tests in ophthalmology, cardiology, and dermatology^1,4–6^. Especially in ophthalmology, OCT has become one of the most valuable imaging tools for diagnosing eye diseases^7–9^. In ophthalmic OCT application, wavelengths of 800 nm, 1000 nm, and 1300 nm are mostly used as light sources^3,7,10,11^. Although 1300-nm OCT is used for dermatological, cardiological, and corneal imaging, the high water absorption feature of the vitreous body of the human eye limits the depth of retinal OCT imaging. The 800-nm OCT system provides more detailed morphological information, microvascular mapping of the retina with a relatively high axial resolution, and it has been successfully popularized in the clinic setting. However, it is difficult to image deeper posterior areas of the eye, such as the choroid region, because of the strong absorption and reflection of the retinal pigment epithelium (RPE) and highly pigmented choroidal melanocytes. Considering the scattering and absorption properties of the eye, the 1000-nm OCT system is preferred for retinal imaging^10^.

In order to successfully apply a biomedical imaging modality to the clinical setting, it must be non-invasive, non-labeling, real-time, *in-vivo* imaging with high-resolution, a sufficient imaging range, and short acquisition time. Therefore, the 1000-nm OCT system needs to have adequate imaging speed (more than 100 kHz) and imaging depth range to capture the deeper choroid layer, including the microvascular structure. One of the high-speed OCT systems is the spectral domain OCT (SD-OCT) system, which uses a broadband light source, such as a superluminescent diode, and a spectrometer to measure the optical interference signals. However, with the SD-OCT system, the imaging speed and imaging depth range are limited by the characteristics of the spectrometer^12^. Silicon-based spectrometers are widely used for the SD-OCT system; however, this device has a low sensitivity at a 1000-nm wavelength than at an 800-nm wavelength. Therefore, an InGaAs CCD camera can be used for a 1000-nm SD-OCT system; however, it is not cost effective, and it has a low pixel number, making it difficult to obtain an OCT image of the deeper retinal layer.

Another high-speed OCT system is swept-source OCT (SS-OCT), which uses a wavelength-swept laser as the light source. SS-OCT systems have advantages of a high imaging speed and longer imaging depth range with a simple and cost-effective detecting scheme (single photon detector) compared with other OCT systems, such as time domain-OCT and SD-OCT^7,11,13–17^. The characteristics of SS-OCT systems mainly depend on the performances of the wavelength-swept source. Many kinds of wavelength-swept sources have been reported, and they tune a lasing wavelength based on mechanical movements of a wavelength-tunable component in the laser cavity. The sweeping behaviors are determined by the mechanical movements of the wavelength-tunable components, such as a the Fabry-Perot tunable filter^18,19^, a polygon mirror scanner^20^, tunable microelectromechanical system filter with a semiconductor optical amplifier (SOA)^21^, or vertical cavity surface-emitting laser^7,16^.

This mechanical movement requires depletion and accumulation of momentum for its completion, which can be affected to hysteresis or experience undesirable drifts of operational scheme, such as thermal drift because of friction. Unstable or drifting mechanical movements can lead to overall degradation of source performance^11,14,15,22^.

Recently, Insight Photonic Solutions, Inc. (Lafayette, CO, USA) reported an akinetic wavelength-swept laser with an integrated semiconductor opto-electronic design without the need for external cavity coupling at the center wavelength of 1060 nm, 1310 nm, and 1550 nm^14,15,22^. The company successfully showed an SS-OCT image of the human retina, skin, and tooth and an OCT angiogram of human skin owing to the phase-stable swept source. However, the swept source contains valid and invalid points that can limit available sampling points and cause data acquisition to be complex. Therefore, a k-clock has to be used to synchronize acquisition of the DAQ card with a delay on the start of the sweep in order to remove invalid data points^15^, and only 780 sample numbers are used for OCT images, which can affect the image quality.

Another type of akinetic wavelength-swept laser is based on the active mode locking (AML) method with dispersive fiber cavity^11,23–33^. Its feasibility for use with fiber Bragg grating sensor systems^25^ and SS-OCT systems^11,23,26–33^ with AML wavelength-swept sources has been reported. The SS-OCT images showed various samples, such as cover glasses, adhesive tape, and an *in-vivo* human finger, at a sweep rate of 1 kHz. In addition, the *in-vivo* OCT image of a human cornea was reported at a sweep rate of 100 kHz by directly modulating the gain medium with the sinusoidal signal^11^. Although the mechanical tunable filters are successfully eliminated with AML methods, the main limitation of *in-vivo* OCT imaging remains: a short image depth range (coherence length of the laser). Therefore, in a previous study, a frequency shifter was inserted into the interferometer to extend the imaging depth range, but the whole corneal structure still could not be shown simultaneously^11^. In addition, the center wavelengths of all studies were 1300 nm and 1550 nm, which are unsuitable for *in-vivo* human retinal OCT imaging^11,23,26^.

In the present study, we describe the use of a pulse-modulated AML wavelength-swept fiber laser with a 1080-nm wavelength for *in-vivo* human retinal OCT imaging. An electro-optic intensity modulation was inserted to the laser cavity which has 3 dB bandwidth of 20 GHz for the high speed pulse modulation of 50 ps duration. A ring laser cavity, with a length of ~1.542 m (in air), was constructed with all polarization maintain fiber to enhance environmental resistance. The average linewidth of 0.0625 nm and the spectral bandwidth of 81.85 nm were obtained in the static AML condition. The sweeping behavior was determined by a fully electrical signal without a mechanical tuning mechanism. Therefore, the swept laser was driven with a uni-directional linear sweep mechanism with a duty ratio of ~90%. Therefore, we extracted a fully synchronized sweep spectrum without observing a difference between forward and backward sweeping. The -6 dB roll-off of 2.5 mm and -20 dB roll-off of 9 mm were obtained at swept rate of 100 kHz for the SS-OCT system. We successfully obtained *in-vivo* retina images of a human using the OCT system without the additional k-clock and the frequency shifter providing wide field of view (FOV) of 43.1°. The main retina layers such as internal limiting membrane, retinal nerve fiber layer, photoreceptor layers, RPE, choroidal stroma, were clearly distinguished through the OCT image with axial resolution of 6.3 μm.

## Results

### Pulse-modulated active mode locking wavelength-swept laser

**Figure 1:**
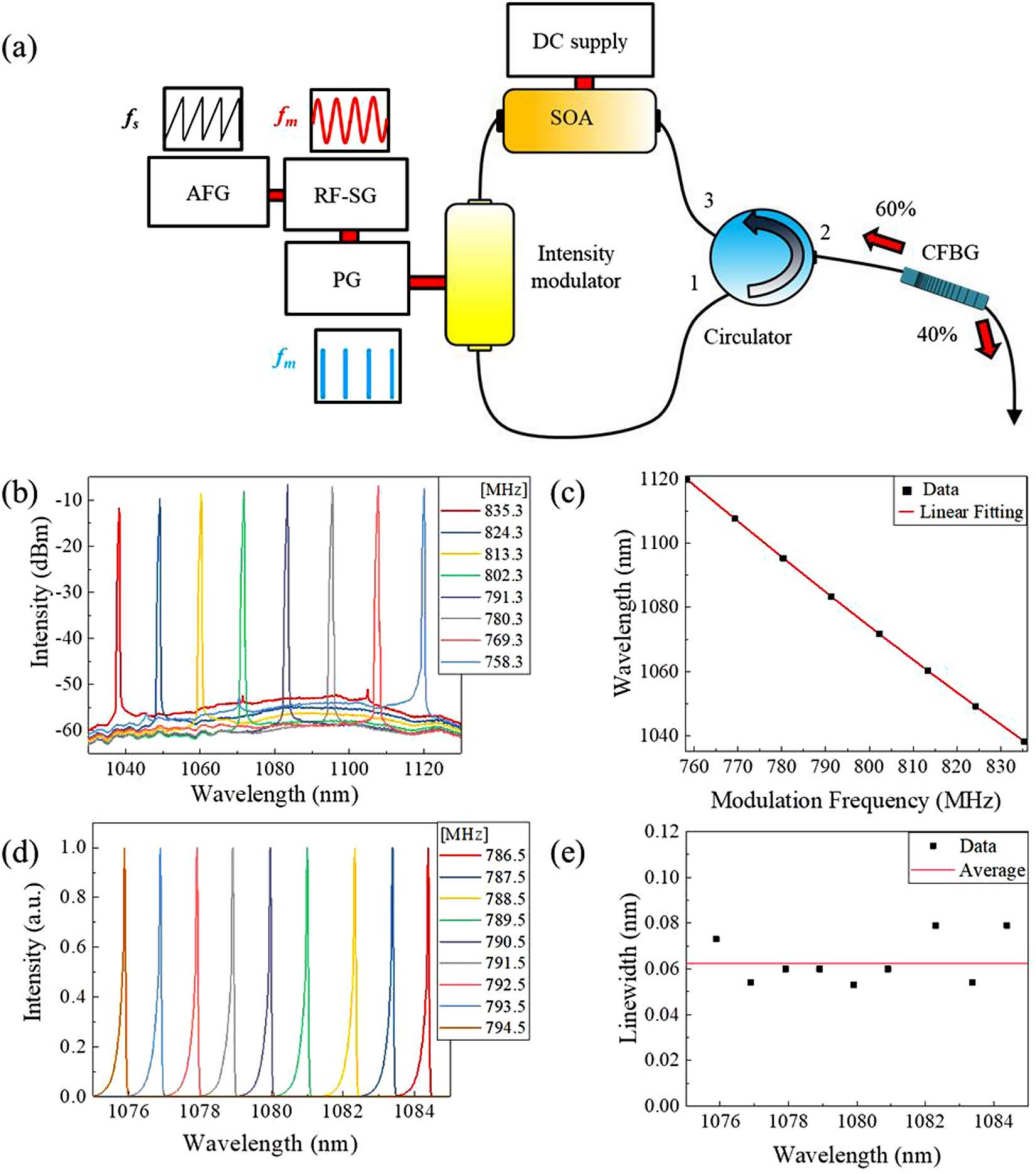
(**a**) Experimental set up of a pulse-modulated active mode locking wavelength-swept fiber laser. AFG: arbitrary function generator, RF-SG: radio-frequency signal generator, PG: pulse generator, SOA: semiconductor optical amplifier, CFBG: chirped fiber Bragg grating (**b**) Static output spectrum with discrete variation of modulation frequency (758.3 MHz–835.3 MHz) in log scale. (**c**) Relationship between RF modulation frequency (*f*_*m*_) and output wavelength (*λ*_*m*_) of the pulse-modulated AML wavelength-swept fiber laser (**d**) Static output spectrum with discrete variation of modulation frequency (786.5 MHz–794.5 MHz) in linear scale. (**e**) Measured linewidth of the AML fiber laser (Fig. 1d).

Figure 1a shows the experimental setup of a proposed pulse-modulated AML wavelength-swept fiber laser at a 1080-nm wavelength range for *in-vivo* human retinal OCT imaging. The laser cavity consisted of an SOA, optical circulator, and chirped fiber Bragg grating (CFBG) and electro-optic intensity modulators. The electro-optic intensity modulator was inserted for high-speed pulse modulation because the commercial SOA has a switching time larger than 500 ps. All components were made of a polarization-maintaining fiber to protect polarization-dependent properties and ensure environmental resistance. A radiofrequency (RF) signal from an RF signal generator was connected to the pulse generator. The pulse signal with 50 ps pulse duration was used to modulate the gain and loss of the laser cavity in order to achieve the AML condition synchronized with RF signal generator. An arbitrary waveform from an arbitrary function generator (AFG) was used to electrically vary the center frequency of the RF signal generator by using the frequency modulation (FM) function. The optical components were spliced as short as possible to provide a better sweeping property. The total cavity length was 1.542 m (in air), which represents a free spectral range (FSR) of 132.89 MHz. The FSR of the laser cavity is expressed as follows:

1 $$FSR=\frac{c}{{n}_{eff}L}$$

where *c* is the speed of light in the vacuum, *n*_*eff*_ is the effective refractive index, and *L* is the cavity length.

In a harmonic mode-locking condition in a fiber laser cavity, the lasing wavelength, *λ*_*m*_, is determined by the modulation frequency *f*_*m*_ of the applied voltage into the electro-optic intensity modulator with an RF pulse, which is an integer (*N*, mode-locking order) multiplied by the FSR, as follows.

2 $${f}_{m}=N\cdot FSR$$

The zero-order FSR, *FSR*_0_, is given by the cavity length and speed of light in the fiber cavity: *FSR*_0_ = *c*_0_*/nL*. However, in a chromatic dispersive medium, the FSR becomes a function of the wavelength (or wavenumber). The relationship between the lasing wavelength and modulation frequency can be expressed as follows^11^:

3 $${\rm{\Delta }}{\lambda }_{m}=-\,S{\rm{\Delta }}{f}_{m}$$

where Δ*λ*_*m*_ is the deviation in the lasing wavelength, *S* is the tuning sensitivity, and Δ*f*_*m*_ is the deviation of the modulation frequency. The sensitivity is defined as follows:

4 $$S=\frac{{n}_{eff}L}{c{D}_{total}{f}_{m0}}$$

where *n*_*eff*_ is the effective refractive index, *D*_total_ is the dispersion parameter, and *f*_*m*0_ is the modulation frequency^11^.

**Figure 2:**
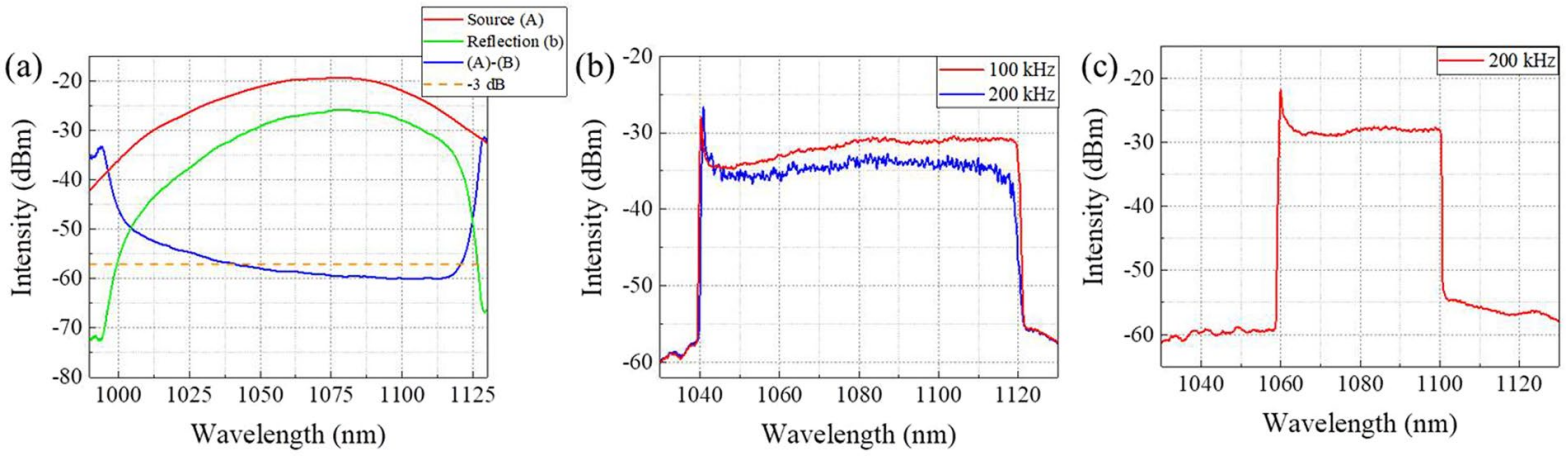
(**a**) Reflection property of the chirped fiber Bragg grating (CFBG) (**b**) OSA peak-hold mode spectra of the pulse-modulated AML wavelength-swept fiber laser output at the *f*_*s*_ = 100 kHz, 200 kHz (**c**) OSA peak-hold mode spectra of the pulse-modulated AML wavelength-swept fiber laser output at the *f*_*s*_ = 200 kHz with spectral bandwidth of 40.07 nm.

Figure 1b shows the static output spectra of the pulse-modulated AML wavelength-swept fiber laser with discrete variation of the modulation frequency in the whole wavelength tuning range. As modulation frequency *f*_*m*_ varied from 758.3 MHz to 835.3 MHz (*N* = 6), the output wavelength *λ*_*m*_ changed from 1120.15 nm to 1038.3 nm. Figure 1c shows the relationship between RF modulation frequency (*f*_*m*_) and output wavelength (*λ*_*m*_) of the pulse-modulated AML wavelength-swept fiber laser. The red line in Fig. 2a is the first-order fitting of the measured data. The measured R-square values of the first-order fitting and residual sum of square were 0.99949 and 2.95549, respectively, which show a high linear relationship according to equations (Eqs 3 and 4). The experimentally measured sensitivity parameter *S* was 1.064 nm/MHz. The theoretical value of the sensitivity was 1.056 nm/MHz according to Eq. 4.

A total tuning range of 81.85 nm was obtained with the center wavelength of 1079.23 nm. The theoretical maximum tuning range can be expressed as follows:

5 $${\rm{\Delta }}{\lambda }_{{\max }}=\frac{1}{|{D}_{total}|{f}_{m0}}$$

It was determined as 139.4 nm based on this equation. In this study, the tuning range of the AML wavelength-swept fiber laser was limited by the gain bandwidth of the SOA and CFBG in the cavity. Figure 2a shows the reflection property of the CFBG. The tuning bandwidth of the laser was primarily limited by the 3-dB reflection bandwidth of the CFBG and optical circulator. The tuning range can be improved by using the wider band SOA, optical circulator, and CFBG.

With the pulse-modulated AML wavelength-swept fiber laser, the relatively wide tuning range without linewidth broadening could be obtained in the whole wavelength range, unlike with direct sinusoidal modulation. Since the wavelength components are almost continuously existed in different time in the laser cavity according to the dispersion, the RF signal should select wavelength component in the short time for narrow linewidth. In addition, with the direct sinusoidal modulated AML laser, a high DC current above the gain threshold should be applied to the SOA before RF modulation to achieve a wide tuning range. This high DC current caused a relatively high-amplified spontaneous emission (ASE) signal, which can also affect the linewidth broadening at the wavelength of the high-gain region. Therefore, the DC current should be controlled to preserve a narrow linewidth, which limits wider tuning bandwidth^11^. Figure 1d shows the static output spectrum with discrete variation of the modulation frequency (786.5–794.5 MHz) at the narrow wavelength tuning range (1084.39–1075.88 nm) in the linear scale. As shown in Fig. 1d, the spectral shapes of the output laser showed a stable and similar tendency, indicating stable lasing through all wavelengths, unlike with direct sinusoidal modulation (Supplementary Figure S1). The measured 3-dB linewidth of the lasing peak was 0.053 nm at the wavelength of 1079.90 nm, which is lower than that of the direct sinusoidal modulated AML wavelength-swept laser. The average 3-dB linewidth of the sinusoidal-modulated (Supplementary Figure S1) and pulse-modulated AML laser (Fig. 1e) was measured as 0.29 nm and 0.0625 nm, respectively. The measured output power of pulse-modulated AML fiber laser was 2.1 mW at 1079.90 nm.

### Sweeping properties of the pulse-modulated AML wavelength-swept laser

With the wavelength-swept laser, a lasing property could be preserved until the sweep rate reached a single round-trip frequency. The single round-trip frequency was defined as follows^19^:

6 $${f}_{single \mbox{-} roundtrip}=\frac{\delta \lambda \cdot c}{{\rm{\Delta }}\lambda \cdot {\rm{L}}\cdot {\rm{n}}}$$

**Figure 3:**
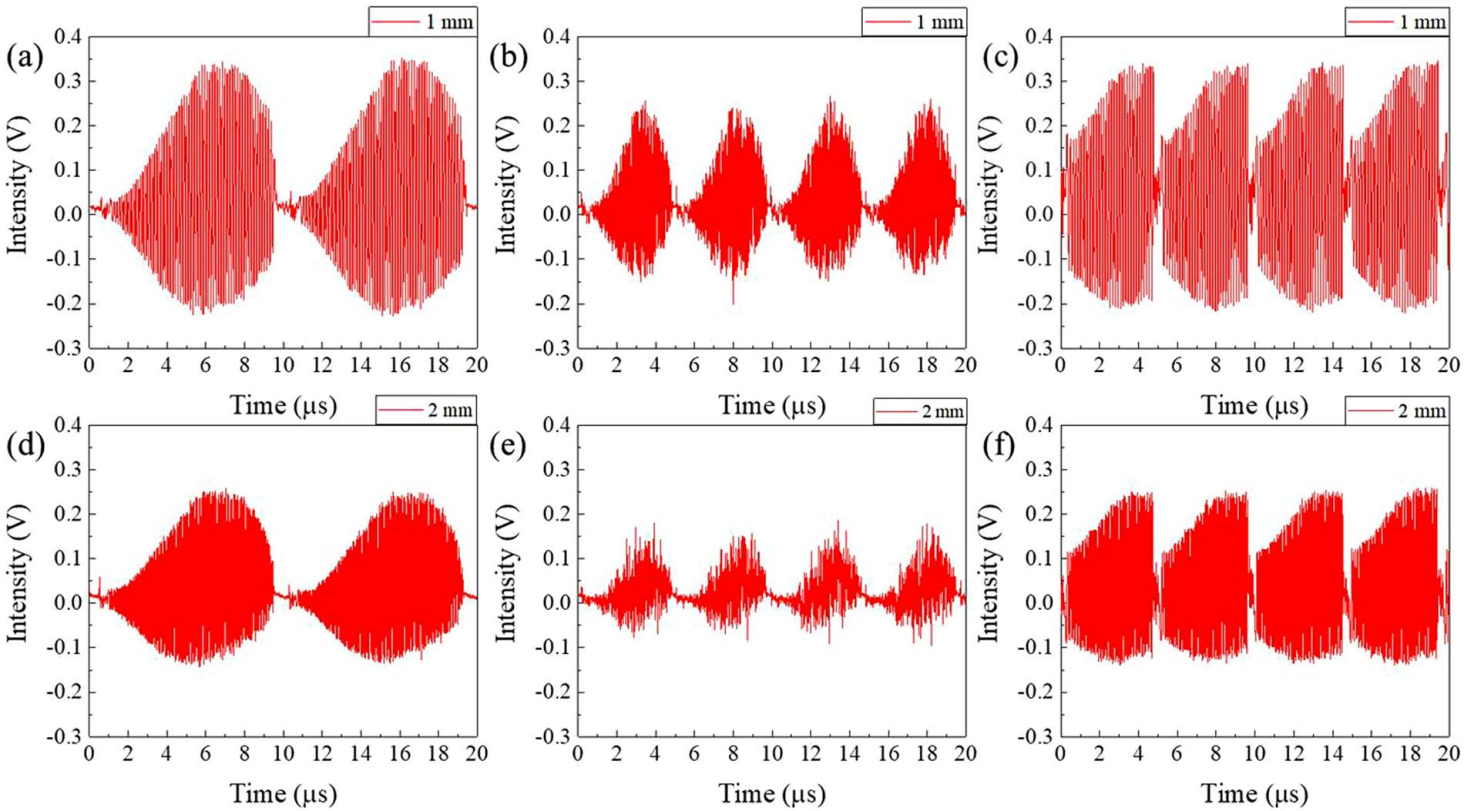
Interferogram of the AML wavelength-swept laser with optical path difference of 1 mm at the condition of (**a**) *f*_*s*_ = 100 kHz, Δλ = 81.15 nm. (**b**) *f*_*s*_ = 200 kHz, Δλ = 81.15 nm. (**c**) *f*_*s*_ = 100 kHz, Δλ = 40 nm. Interferogram of the AML wavelength-swept laser with optical path difference of 2 mm at the condition of (**d**) *f*_*s*_ = 100 kHz, Δλ = 81.15 nm. (**e**) *f*_*s*_ = 200 kHz, Δλ = 81.15 nm. (**f**) *f*_*s*_ = 100 kHz, Δλ = 40.07 nm.

In this experiment, the sweep rate *f*_single-roundtrip_ for a single round trip was about 102.22 kHz, where *δλ* = 0.0625 nm, *c* = 3 × 10^8^, *Δλ* = 81.25 nm, *L* = 1.542 m, and *n* = 1.464. Although wavelength sweeping could be operated at more than 1 MHz, the sweep rate had to be below this frequency to obtain sufficient gain for the lasing. Figure 2b and c show the sweep spectra of the pulse-modulated AML wavelength-swept fiber laser with various sweep rates, *f*_*s*_. The spectra were measured by a peak-hold mode of an optical spectrum analyzer. During wavelength sweeping, the ramp (uni-directional) waveform of the AFG was applied to the RF signal generator with FM function of the RF generator (Fig. 1a). As shown in Fig. 2b, the optical output power was decreased at a sweep rate of 200 kHz because the sweep rate was higher than *f*_single-roundtrip_. To preserve output power at a sweep rate of 200 kHz, the sweep bandwidth had to be half of 100 kHz. In addition, the output power and other laser properties simultaneously degraded, such as the optical linewidth. Figure 3 shows the interferogram of the AML wavelength-swept laser at different conditions when the optical path differences were 1 mm (3a, b, c) and 2 mm (3d, e, f). Compared with the 100-kHz sweep rate, the coherence property was highly degraded at the sweep rate of 200 kHz with the same spectral bandwidth of 81.15 nm, as shown in Fig. 3b and d. For the same laser cavity configurations such as the cavity length, linewidth, gain, the operating spectral bandwidth should be narrower in the case of a 200-kHz sweep rate. Figure 3c and f show the interferogram of the AML wavelength-swept laser at the sweep rate of 200 kHz and operating spectral bandwidth of 40.07 nm (Fig. 2c). By dividing the optical spectral bandwidth in half (100 kHz), the other sweep properties were well preserved at the sweep rate of 200 kHz. In order to use the wide spectral bandwidth at a higher sweep rate, the cavity length should be as short as possible. A wider spectral bandwidth at a higher sweep rate with sufficient lasing properties are expected to be available when the length of the laser cavity is decreased further by using improved fiber splicing techniques.

Here, we can confirm that the duty ratio of the laser is almost 90% because we applied the single-directional drive function thanks to removal of the mechanical tunable filter, which can remove non-linear properties of bi-directional sweeping. A sampling rate of the SS-OCT system, *f*_*sampling*_, can be defined as follows:

7 $${f}_{sampling}=\frac{{f}_{s}\cdot {N}_{sample}}{{R}_{duty}\,}$$

where *f*_s_ is the sweep rate, *N*_*samp*le_ is the sample number for every A-scan and *R*_*duty*_ is the duty ratio. The higher sample number can be achieved at the higher high duty ratio, which makes it possible to obtain better imaging qualities. A conventional mechanical swept laser has an effective duty ratio less than 50% in order to achieve single-directional sweep modes, which require a higher speed digitizer or lower sample number. Therefore, a buffering method is conventionally used for common SS-OCT systems to achieve the duty ratio of about 90%^20,21,34,35^. An advantage of our system is that the entire spectrum is single-directionally swept without the use of a buffering method.

### Characteristics of the swept-source optical coherence tomography system

**Figure 4:**
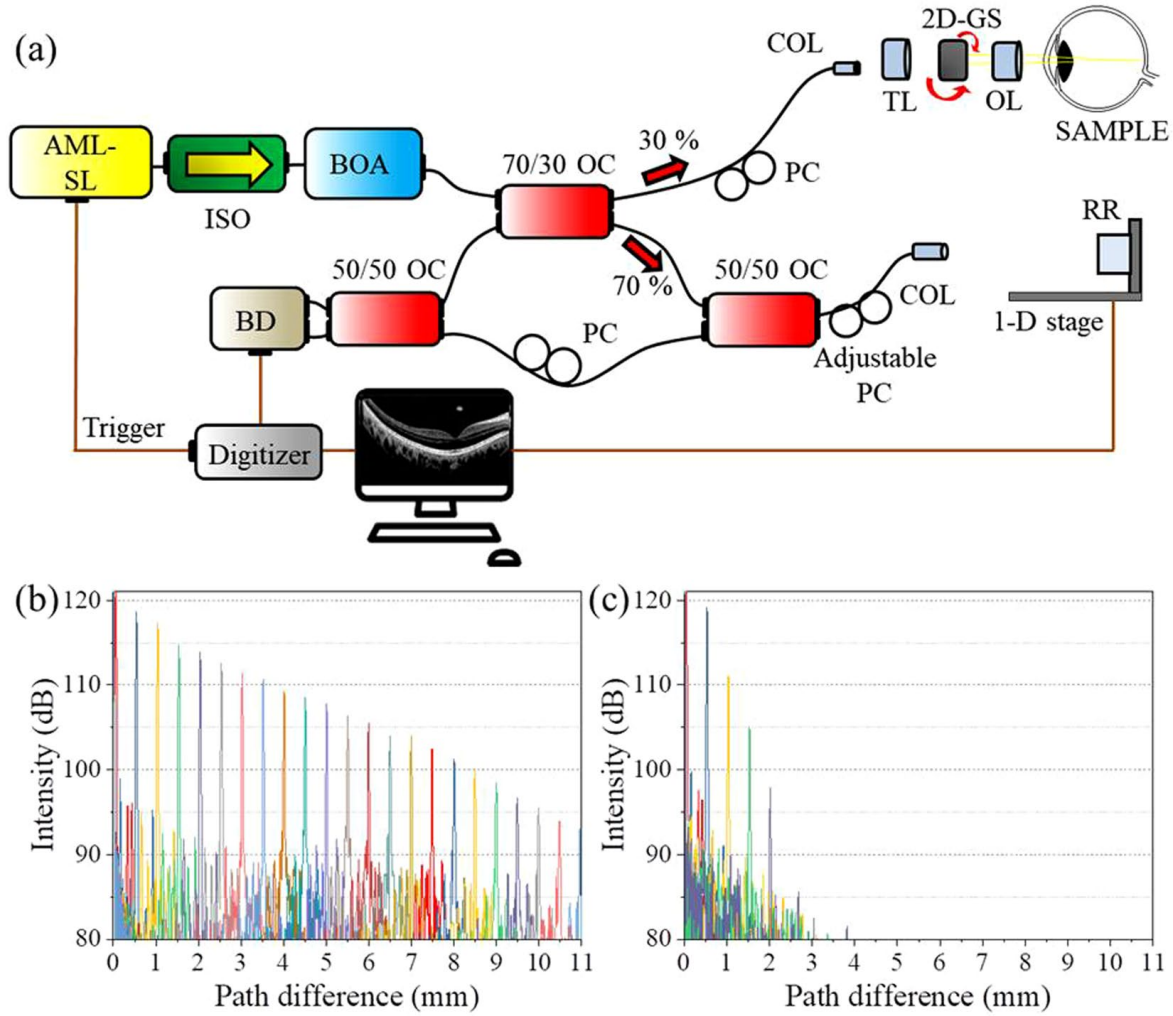
(**a**) The experimental set-up of the SS-OCT system based on the pulse-modulated AML wavelength-swept fiber laser. AML-SL: active-mode-locking swept-laser; PC: polarization controller, ISO: isolator, BOA: booster optical amplifier, OC: optical coupler, 2D-GS: two dimensional galvano scanner, BD: balanced-detector, COL: collimator, OL: optical lens, TL: tube lens, RR: retro reflector (**b**) Roll-off measurement of the pulse-modulated AML wavelength-swept laser with forward sweeping (**c**) Roll-off measurement of the pulse-modulated AML wavelength-swept laser with backward sweeping.

Figure 4a show the experimental setup of the SS-OCT system based on the pulse-modulated AML wavelength-swept fiber laser. For the best OCT imaging with stable laser conditions and data processing capacity, we selected an optimal sweep rate of 100 kHz for the pulse-modulated AML wavelength-swept fiber laser, with an output power of 18.2 mW after the booster optical amplifier. The measured spectral bandwidth of the laser was 81.15 nm. Therefore, according to Eq. (8), the related OCT resolution was theoretically 6.3 μm in air^36^.

8 $${\rm{\delta }}z=\frac{{\rm{2ln2}}}{\pi }\frac{{\lambda }_{0}^{2}}{n\Delta \lambda }$$

The SS-OCT system was configured with a Michelson interferometer. To analyze the OCT performance of the pulse-modulated wavelength-swept AML fiber laser, we measured the point-spread function (PSF) roll-off over the imaging depth in OCT application. For better coherence properties during the sweeping, the laser should be swept from a short to a long wavelength region (forward sweeping). When the optical pulse passes through the gain medium (e.g. SOA) of laser cavity, the optical redshift is induced by the self-phase modulation. This red shifting from modulation of the gain medium allows stable pulsation for short to long wavelength tuning^37^. In contrast, the coherence properties were degraded for backward sweeping (long to short wavelength region). To examine this experimentally, two types of waveforms from an AFG were applied to the laser. One was a forward sweeping, and the other was a backward sweeping with single-directional linear drive function. The characteristics of these waveforms were analyzed and compared. Figure 4b and c show the PSF roll-off of the AML wavelength-swept fiber laser for forward sweeping and backward sweeping, respectively. As expected, the PSF signals were significantly decreased in backward sweeping which showed almost meaningless results at >1 mm (Supplementary Figure S2). In forward sweeping, the total 20-dB roll-off imaging range of 9 mm was obtained which is almost 5 times higher than that of sinusoidal modulation. The measured sensitivity of the system was 119.8 dB. The proposed laser is basically a pulse laser, therefore, the pulse duration can affect a fringe visibility as well as roll-off of the OCT system. In term of the coherence behavior, the pulse width is one of the factors which could affect the fringe visibilities because of the time mismatch of the time-delayed pulses pair from reference and sample arms. However, the 20-dB roll-off of 9 mm is enough to image the human retina since the structural thickness and image range of a posterior eye is around 3 mm. In our previous study, we inserted a frequency shifter to enhance the imaging depth range because of the low coherence length of the sweeping source^11^. However, in this study, we obtained enough imaging range for *in-vivo* retina OCT imaging thanks to the linewidth enhancement of the pulse modulation. Additionally, the OCT system could be operated with a sweep rate of 200 kHz when the spectral bandwidth was decreased to 40.07 nm (Supplementary Figure S3).

### *In-vivo* optical coherence tomography image based on the pulse-modulated AML wavelength-swept laser

**Figure 5:**
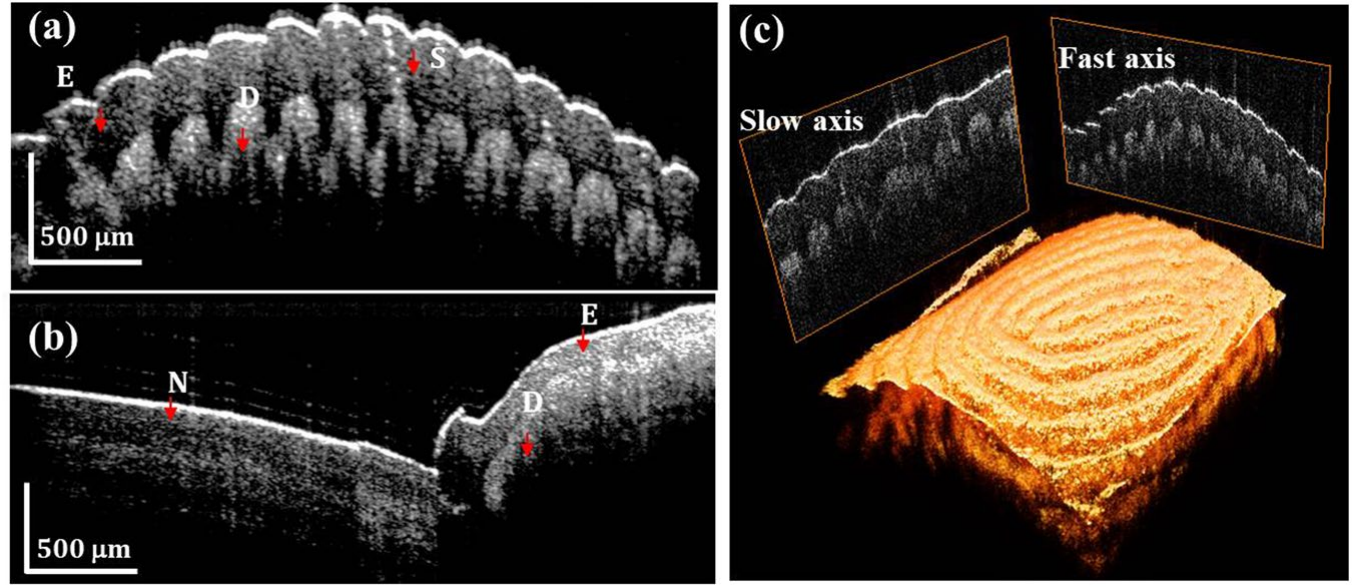
*In-vivo* SS-OCT images of human fingers (**a**) 2D cross-sectional image of human finger. (**b**) 2D cross-sectional image of human finger: nail and finger boundary. (**c**) 3D rendered OCT image of human finger; E: epidermis, S: sweat duct, D: dermis, N: Nail.

Using the proposed SS-OCT system, we firstly obtained *in-vivo* OCT images of a human finger. The measured transverse spot size on the sample was 20 µm, and the measured axial resolution was 6.3 μm. The average incident power on the finger surface was 1.8 mW, which is below the American National Standards Institute (ANSI) limit of 1.925 mW at a wavelength of 1060 nm^38^. Figure 5a and b show the 2-dimensional (2D) cross-sectional image of a human finger and nail/finger boundary, and Fig. 5c shows the 3-dimensional (3D) OCT image of the human finger (Supplementary Video S1). Because of the longer imaging range and high axial resolution of the system, the high contrast OCT image could be obtained with the pulse-modulated AML wavelength-swept laser compared with the sinusoidal-modulated AML wavelength-swept laser.

**Figure 6:**
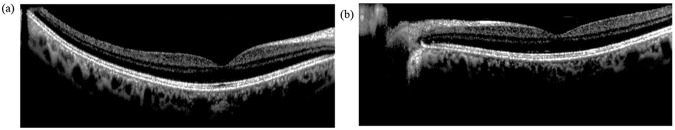
*In-vivo* cross-sectional SS-OCT images of the human retina (**a**) 100 times averaged, 43 years old, left eye, male (Patient A) (**b**) 100 times averaged, 43 years old, right eye, male (Patient A).

**Figure 7:**
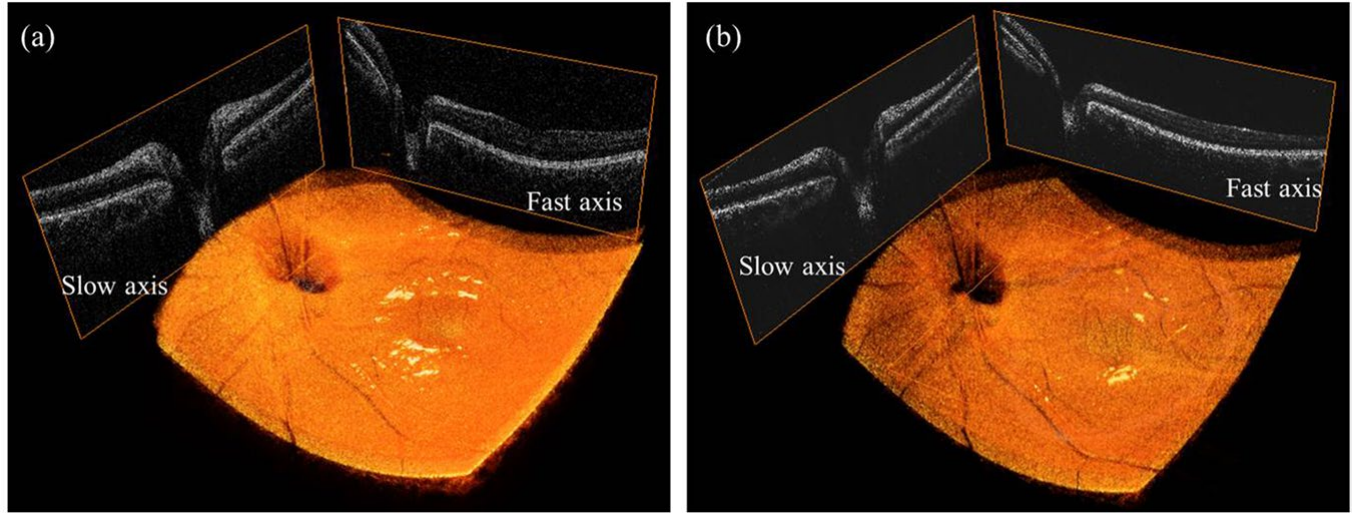
(**a**) *In-vivo* 3D rendered OCT image of human retina (Patient A) (**b**) *In-vivo* 3D rendered OCT image of human retina (Patient B).

For retinal OCT imaging, the optical lens at the sample arm was removed, and the light was focused by the eye of a patient. The average incident power on the eye surface was 1.3 mW, which is less than the ANSI limit of 1.925 mW at a wavelength of 1060 nm^38^. Figure 6a and b show the cross-sectional OCT image of the human retinas of the left and right eyes, respectively. The main layers of the retina, such as the internal limiting membrane, retinal nerve fiber layer, photoreceptor layers, RPE, choroidal stroma, could be distinguished because of the high axial resolution of the proposed sweep source and the OCT system. The 3D OCT images were created from a Fourier transform of 500 × 500 × 4096 voxels per volume data set. The acquisition times were 5 milliseconds and 2.5 seconds for 2D and 3D OCT images, respectively. Figure 6c shows the enface image that was obtained from the 3D OCT image. Compared with the commercial fundus image at 50° (Supplementary Figure S4), we confirmed that the image range of the retinal OCT system was 43.1°, which can be used to simultaneously observe macular and nerve bundle regions. The 3D OCT images of human retina were obtained after motion compensation^38^ (Fig. 7, Supplementary Videos S2 and S3).

## Discussion

Akinetic wavelength-swept sources have many advantages, such as phase stability, a high duty ratio without a buffering method, linear sweeping, and a high repetition rate. Many studies have reported on the akinetic AML wavelength-swept laser; however, the sweeping performances were insufficient to obtain high-speed *in-vivo* OCT imaging. In addition, the center wavelength range was unsuitable for *in-vivo* retinal OCT imaging because of the high water content of the human eye. We successfully obtained *in-vivo* OCT images of the human retina using a pulse-modulated akinetic AML wavelength-swept fiber laser with the center wavelength of 1080 nm. To enhance environmental resistance, the entire fiber-optic ring laser cavity was constructed with polarization-maintaining fiber instead of single-mode fiber. The pulse modulation suppresses the DC current, which affects the linewidth broadening; therefore, the average linewidth of 0.0625 nm and spectral bandwidth of 81.85 nm were obtained in the static AML condition, showing stable spectral shapes over the tuning range. The proposed wavelength-swept laser has the unidirectional sweeping characteristic of about a 90% duty ratio, which can reduce the sampling rate compared with the commercialized bi-directional swept source under the 60% duty ratio. We successfully obtained *in-vivo* 3D OCT images of human retinas at a sweep rate of 100 kHz without an optical frequency shifter. Further study of the following is needed to improve the system: (1) shortening of the laser cavity length to increase swept performance at the high sweep rate, (2) using high-speed and more stable RF electronics for phase stability (3) optimizing the pulse duration of the laser to enhance the fringe visibility (4) enhancement of the spectral bandwidth using a broadband CFBG and gain medium, (5) applying wider-angle retinal OCT angiography using a laser with a higher sweep rate.

## Methods

### *In-vivo* optical coherence tomography imaging and 3-dimensional image reconstruction

This study was approved by the institutional review board of the Gwangju Institute of Science and Technology (approval number 20180629-HR-36-02-02), and written informed consent was obtained from 2 healthy subjects before conducting the imaging procedure. All experiments were performed in accordance with relevant guidelines and regulations. To obtain the 3D image of the human retina, a subject was positioned on the commercial OCT headrest. The averaged laser power energy on the eye surface was 1.3 mW, which is less than the ANSI limitation of 1.925 mW for a wavelength of 1060 nm^37^. All volumetric data (500 × 500 × 4096 pixels) were rendered using the commercial software Amira (FEI Visualization Sciences Group) after motion compensation^39^.

### Pulse-modulated active mode locking wavelength-swept laser

The SOA (SOA-1060-90-PM-30dB, Innolume Inc.) has a 3-dB bandwidth of 90 nm and center wavelength of 1070 nm when a current of 400 mA is applied. The optical circulator was inserted for ring cavity configuration and used for unidirectional lasing by protecting bi-directional emission of the SOA and optical reflection between the fiber components. The optical circulator (Oz Optics Inc.) has a 3-dB operating bandwidth of 100 nm with a center wavelength of 1060 nm and an isolation more than 20 dB. The CFBG with a dispersion parameter of 9 ps/nm at *λ* = 1060 nm was inserted into the laser cavity to provide chromatic dispersion in the wavelength-dependent AML condition. We used anomalous dispersion for the fiber laser cavity because it can suppress SPM-induced spectral broadening and create a narrower linewidth of the laser^29^. Additionally, the CFBG acts as an optical output coupler in the fiber laser cavity, which has a reflectivity of 60 ± 3%, and the operating spectral bandwidth of the CFBG is about 120 nm. An electro-optic intensity modulator (NIR-MX-LN-20, iXblue Inc.) was inserted to the laser cavity which has 3 dB bandwidth of 20 GHz for the high speed pulse modulation of 50 ps duration. A pulse generator (EPG-210, Alnair Lab) was used to modulate electro-optic intensity modulator with electrical pulse of 50 ps duration which is synchronized with frequency of the RF signal generator.

### Interferogram and PSF measurement

The interferogram and PSF measurement system was constructed based on a Michelson interferometer, which consists of four broadband 50/50 couplers (Supplementary Figure S5). The optical path difference was determined by translating the pair of optical lens (AC254-035-C-ML, Thorlabs) and mirror of one of the interferometer arms. The interference signal was detected by a balanced photodetector (PDB460C, Thorlabs), and 4096 samples were collected by every A-line with a 12-bit, 500 MS/s digitizer (ATS9360, AlazarTech, Inc.). Although the AML wavelength-swept fiber laser can be driven with linear sweeping signal, the system should be calibrated for high wavenumber linearity. The linearization in wavenumber domain was performed with a phase oriented fringe analyzing technique^40,41^. The interference signals were collected for every 0.25 mm optical path difference between the sample and reference arms. The optical phase was extracted from an arbitrary interference signal, and the calibration table was written as look-up table (LUT) to perform the wavenumber linearization in real-time. The maintenances of the PSF correspond to the OPD variation was confirmed. The PSF signals were collected for every 500-μm path difference after calibration.

### Optical coherence tomography system

The swept-source OCT system was constructed based on a Michelson interferometer which consisted of a broadband 70/30 coupler, couple of 50/50 couplers. The average power illuminated on the sample was low as 1.5 mW. The OCT interference signal was detected by a balanced photodetector (PDB460C, Thorlabs) and 4096 samples were collected via every A-line with a 12-bit, 500 MS/s digitizer (ATS9360, Alazartech Inc.).). The interfered signals were linearly sampled without k-clock and the spectrum interpolation for calibration to the k-space was performed on all A-line data prior to the fast Fourier transform (FFT) process. A tube lens is used to control diopter for each patient and the length of reference arm is automatically controlled with range of ±2  mm. An automatically controlled adjustable polarization controller is inserted in the reference arm to enhance image contrast and suppress the polarization dependent noise. The scan range was set as wide as 12 × 12 mm^2^ with an interval of 24 μm for both B- and C-scans such that one 3D OCT image was composed of equally spaced 500 × 500 × 4096 voxels. Considering the sweep rate of the 100 kHz laser, the frame rate of 2D image was 200 fps, and one 3D volume image was acquired within 2.5 s, which is faster than the normal human tear breakup time (TBUT). The axial resolution of the system was measured to be 6.3 μm in air.

## Electronic supplementary material

Supplementary video11

Supplementary video2

Supplementary video3

Supplementary document 
