# Supplementary material for: Akinetic swept-source optical coherence tomography based on a pulse-modulated active mode locking fiber laser for human retinal imaging
Source: Sci Rep. 2018 Dec 5;8:17660. doi: 10.1038/s41598-018-36252-z (PMC6281618; doi:10.1038/s41598-018-36252-z)
Supplement: Supplementary file 4 — Supplementary document [file 41598_2018_36252_MOESM4_ESM.docx]

Akinetic swept-source optical coherence tomography based on a pulse-modulated active mode locking fiber laser for human retinal imaging

Hwi Don Lee^1^, Gyeong Hun Kim^2^, Jun Geun Shin^1^, Boram Lee^3^, Chang-Seok Kim^2*^, Tae Joong Eom^1*^

*^1^ Advanced Photonics Research Institute, Gwangju Institute of Science and Technology, 123 Cheomdan-gwagiro, Buk-gu, Gwangju 61005, South Korea*

*^2^ Department of Cogno-Mechatronics Engineering, Pusan National University, Busan, 46241, South Korea.*

^3^ *Department of ophthalmology, Korea University college of medicine, Seoul, 02841, South Korea.*

^*^ Correspondence and requests for materials should be addressed to C.-S. K. ([ckim@pusan.ac.kr](mailto:ckim@pusan.ac.kr)) or T.J.E. ([eomtj@gist.ac.kr](mailto:eomtj@gist.ac.kr)).

**Supplementary Figures**

**
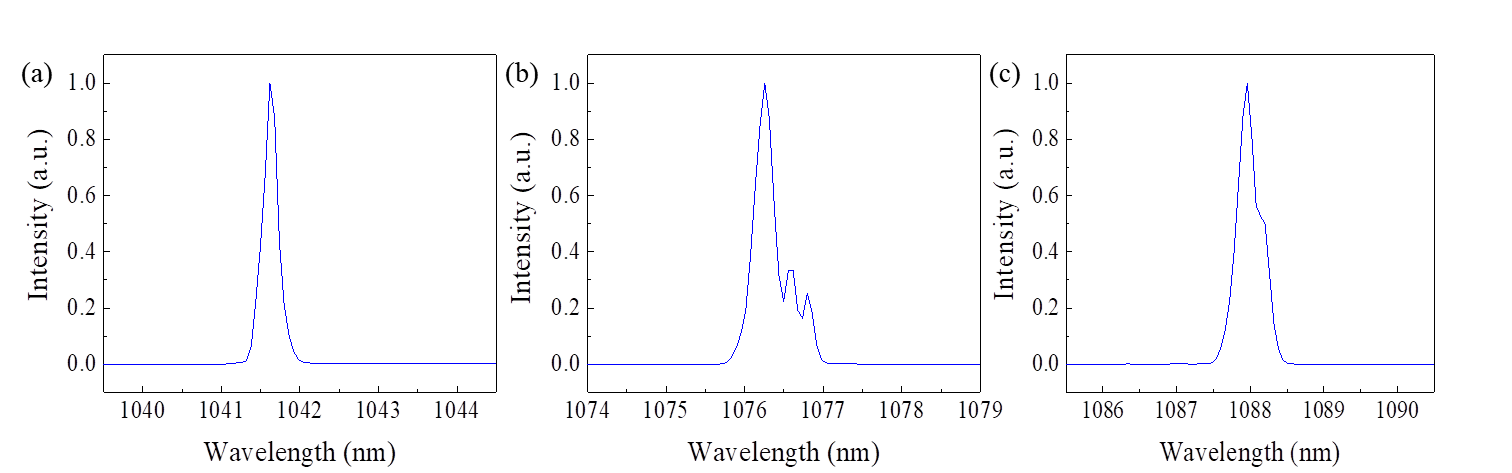
**

**Supplementary Figure S1 | Static spectrum of the sinusoidal AML wavelength swept laser at different center wavelength (a) 1041.6 nm (b) 1076.2 nm (c) 1087.9 nm**

**
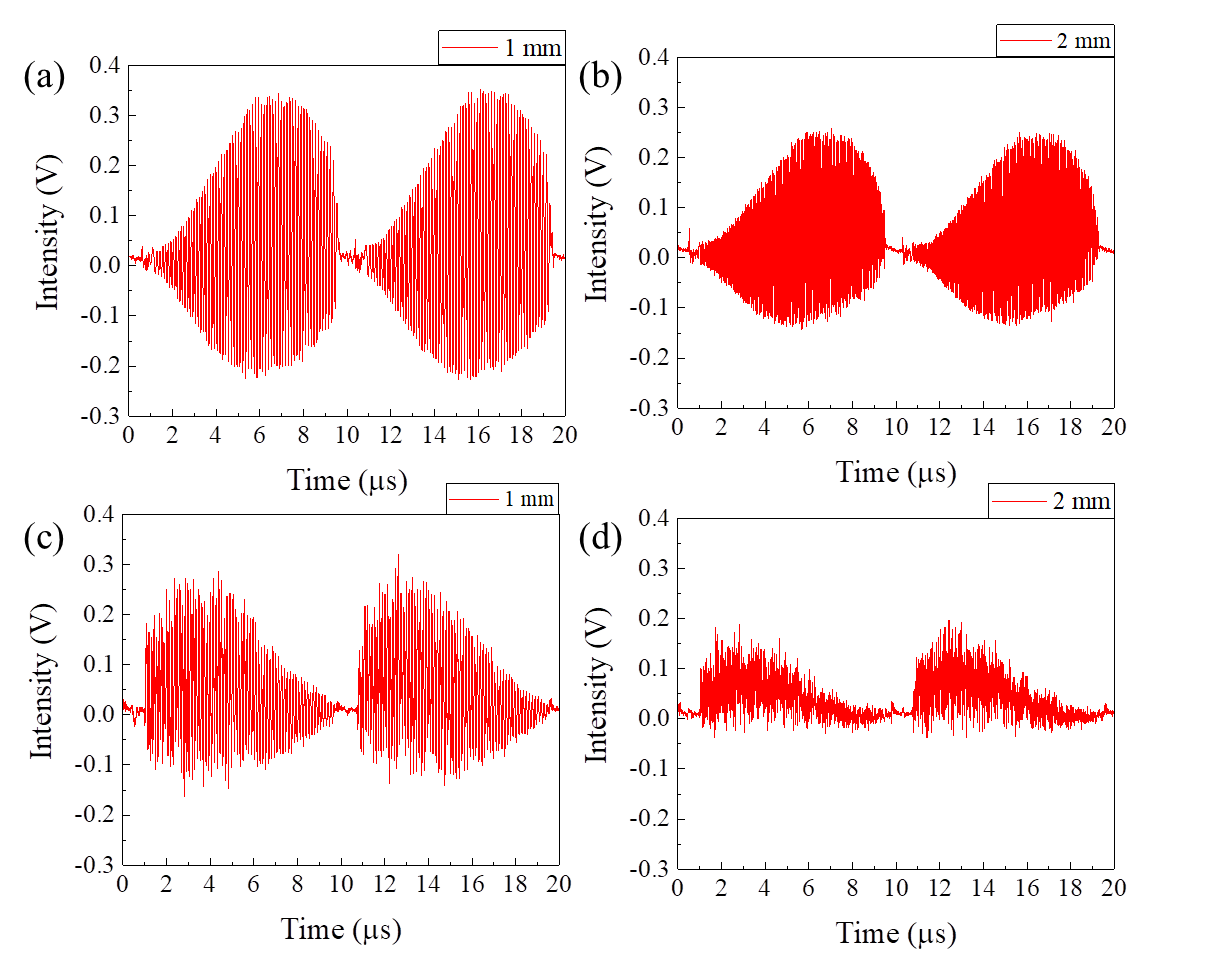
**

**Supplementary Figure S2 | Interferogram of the pulse-modulated AML wavelength-swept laser with different swept direction at swept rate of 100 kHz (a) forward sweeping, path difference = 1 mm (b) forward sweeping, path difference 2 mm (c) backward sweeping, path difference = 1 mm (d) backward sweeping, path difference = 2 mm**

**Supplementary Figure S3 | Point spread function of the pulse-modulated AML wavelength swept laser at swept rate of 200 kHz and swept bandwidth of 40.07 nm**

**
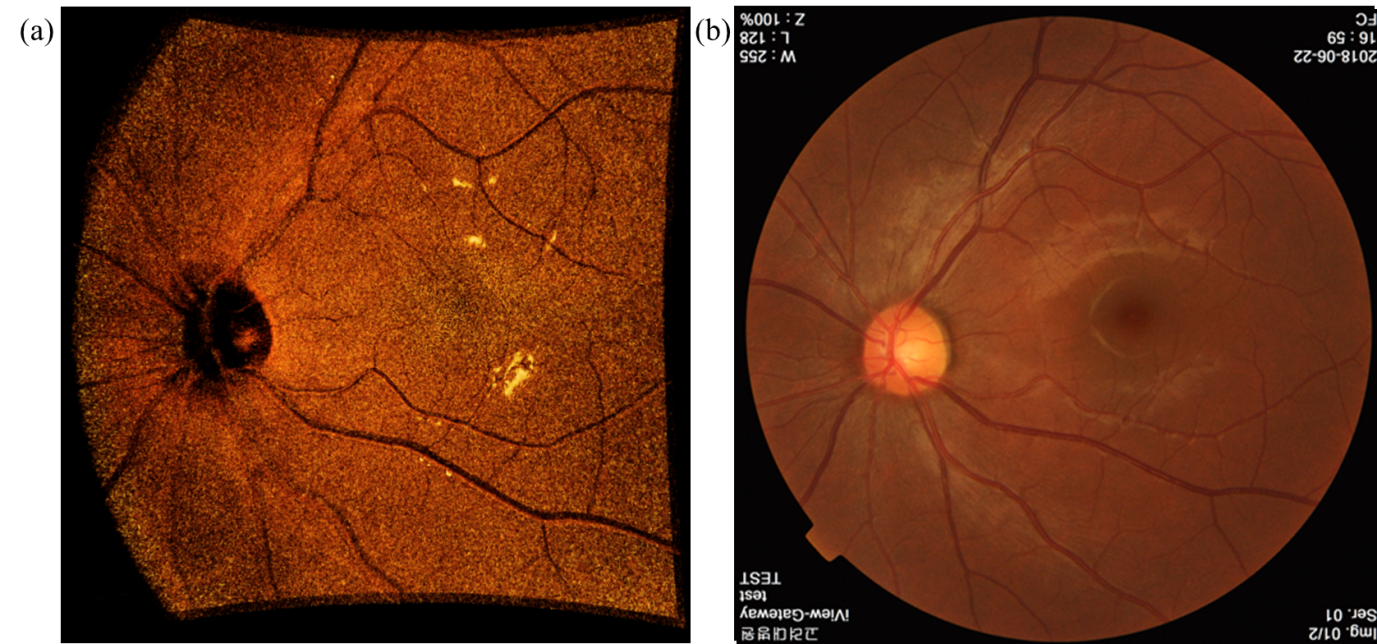
**

**Supplementary Figure S4 | (a) Single shot enface OCT image of the human retina, 27 years old, female.**

**(b) Commercial fundus image of a human retina, 27 years old, female. (Patient B)**

**
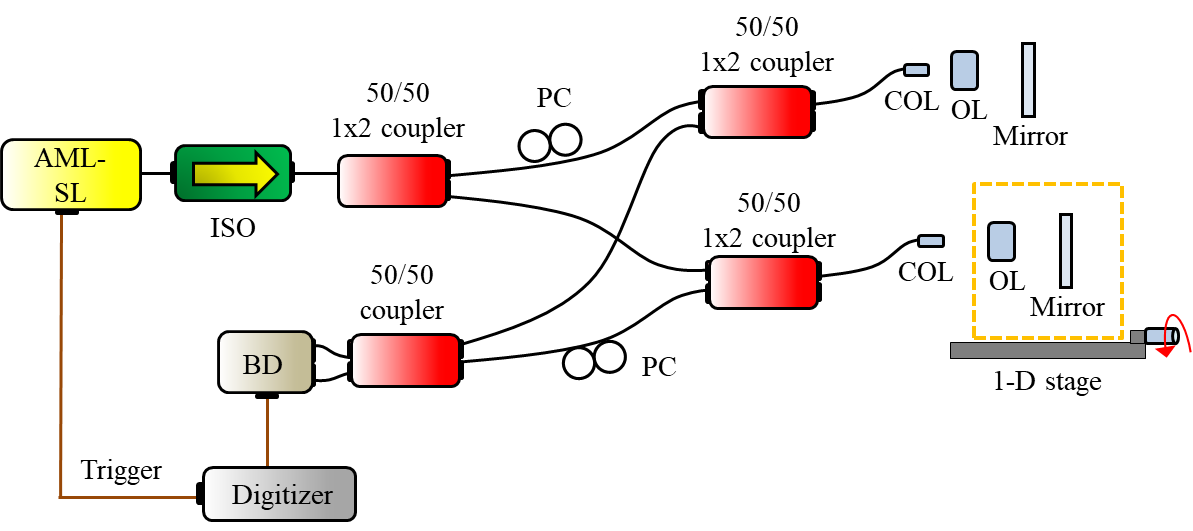
**

**Supplementary Figure S5 | Experimental set up of the interferogram and PSF measurement with proposed pulse-modulated AML wavelength-swept laser.**

**
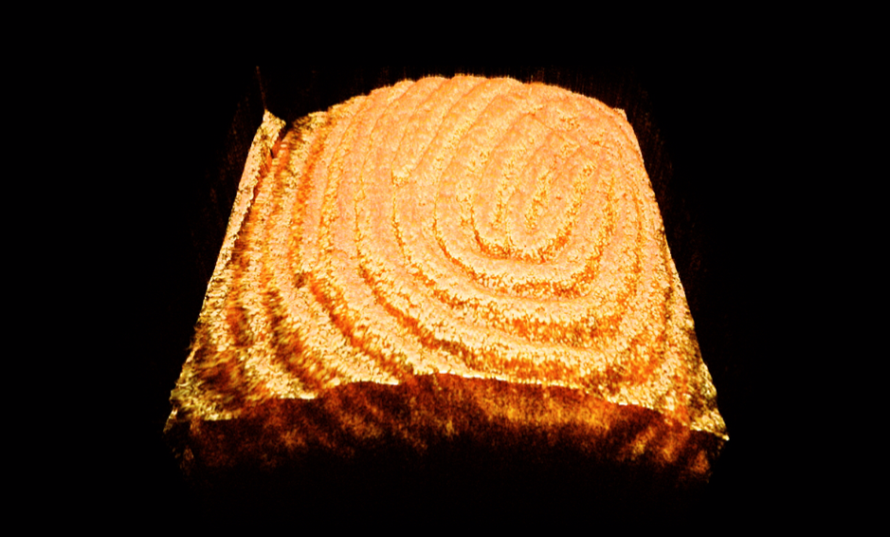
**

**Supplementary Video S1 | *In-vivo* three-dimensional retina imaging movie of the human finger. The movie is processed by commercial software, Amira.**

**
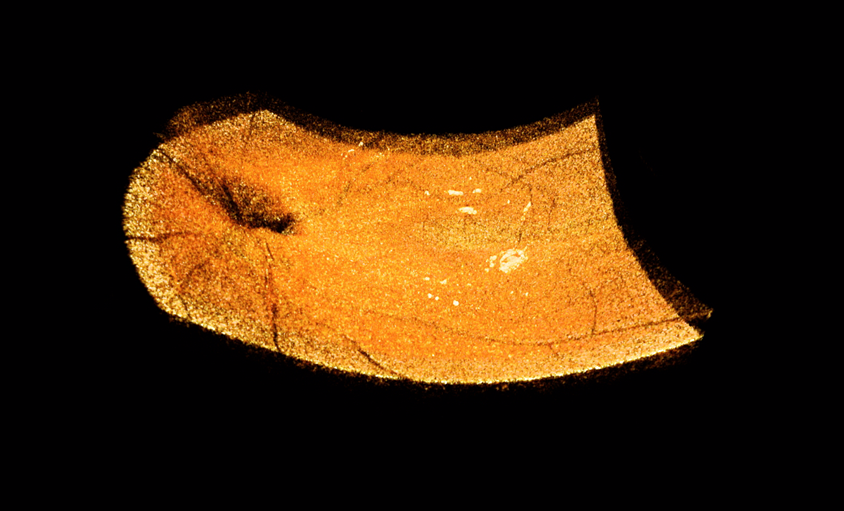
**

**Supplementary Video S2 *| In-vivo* three-dimensional retina imaging movie of the human retina (Patient A). The movie is processed by commercial software, Amira.**

**
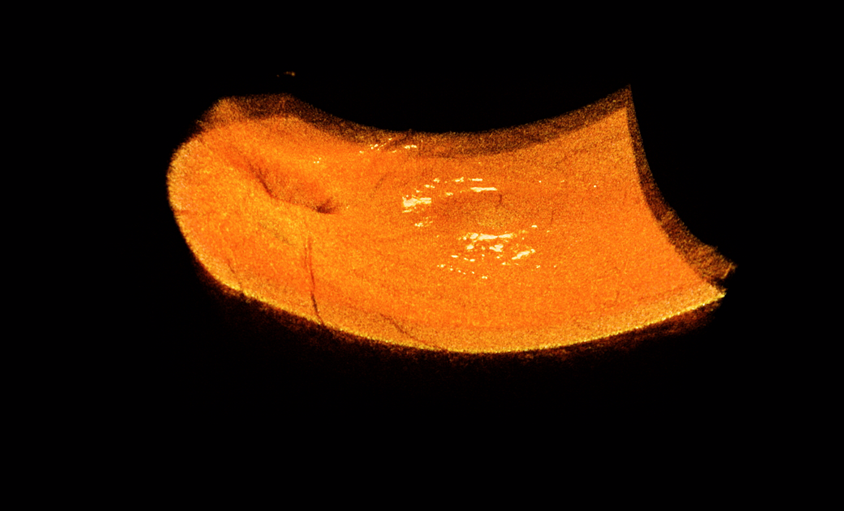
**

**Supplementary Video S3 *| In-vivo* three-dimensional retina imaging movie of the human retina (Patient B). The movie is processed by commercial software, Amira.**
